# Low-Sample Input miRNA Profiling in Extracellular Vesicles-Enriched Plasma Fractions from Older Adults With/out Cognitive Impairment

**DOI:** 10.3390/ijms27177838

**Published:** 2026-09-01

**Authors:** Arianna Consiglio, Ylenia Antonacci, Claudia Lograno, Claudia Chiapparino, Katia Pinto, Apollonia Tullo, Maria Liguori

**Affiliations:** 1 Institute for Biomedical Technologies, National Research Council, Bari Unit, 70125 Bari, Italy; arianna.consiglio@cnr.it (A.C.); ylenia.antonacci@cnr.it (Y.A.); 2 Institute of Biomembranes, Bioenergetics and Molecular Biotechnologies, National Research Council, 70125 Bari, Italy; apollonia.tullo@cnr.it; 3 Alzheimer’s Association Bari, Confederated to Alzheimer’s Federation Italy, 70124 Bari, Italy; logranoclaudia@gmail.com (C.L.); claudiachiapparino@gmail.com (C.C.); katia_pinto@libero.it (K.P.)

**Keywords:** Extracellular Vesicles (EVs), microRNA (miRNA), mild cognitive impairment, circulating biomarkers, aging, neurodegeneration

## Abstract

The RNA cargo of extracellular vesicles (EVs) has been investigated to search for circulating biomarkers possibly associated with a given disease. In this direction, vesicular small RNAs like microRNAs (miRNAs) showed great potential e.g., in monitoring neurodegenerative diseases and their clinical signs. In this exploratory cross-sectional study, we looked for miRNAs possibly associated with age-related cognitive impairment. Using a high processivity and sensitive OpenArray technology, we analyzed the expression of 223 miRNAs in plasmatic total EV-enriched fractions from 43 subjects older than 65 years who received neuropsychological examination. In a smaller cohort of 17 subjects, we verified any miRNA discrepancies between EV-associated and unfractionated plasma samples. A set of candidate miRNAs showed reduced expression in EV-associated fractions from cognitively impaired (n. 12) compared to cognitively preserved (n. 23) subjects. In particular, the expression of 15 of them (miR-106b-5p, miR-128-3p, miR-15a-5p, miR-16-5p, miR-17-5p, miR-191-5p, miR-192-5p, miR-199a-3p, miR-221-3p, miR-222-3p, miR-223-3p, miR-22-3p, miR-26a-5p, miR-335-5p, miR-505-3p) correlated with the MMSE score. Pathways of neurodegeneration (ND), such as PI3K-Akt, Ras/MAPK, and Wnt signaling, resulted significantly involved. Furthermore, 18 miRNAs were implicated in metal ion binding processes, thus supporting the key role of this molecular mechanism in ND-associated cognitive dysfunction. The paired analysis of EV-plasma samples reported the overall upregulation of most of the 223 miRNAs in plasma samples, whereas miR-424-5p, miR-505-3p, miR-192-5p, miR-338-3p and miR-1271-5p were almost stable in the two fractions and miR-205-5p and miR-28-5p were significantly upregulated in EVs compared to plasma. In our view, these findings support the potential relevance of vesicular-associated miRNAs as candidates of age-related cognitive dysfunctions, although for clinical practice some methodological issues may arise, i.e., the feasibility of total EVs extraction. Further confirmations are needed, e.g., the complete characterization of EVs’ cargo and origin, and validation in larger samples.

## 1. Introduction

Extracellular vesicles (EVs) comprise heterogeneous membrane-bound nanoparticles, including exosomes (EXOs) generated within the endosomal system and released into the extracellular space, and microvesicles (MVs) shed directly from the plasma membrane [1]. Confirmed evidence shows that EVs represent an important channel of cellular communication, acting as vehicles among cells for the transfer of cytosolic or membrane proteins, lipids, DNA, and RNA, thus regulating physiological and pathological functions of both target and progenitor cells [2]. Isolated from most of the biological fluids, their content and concentration appear to vary under both physiological and pathological conditions [3,4].

In Central Nervous System (CNS) activities, intercellular communication via EVs—mainly EXOs and MVs—seems to exert an important functional impact in internal homeostasis, especially for myelin formation, metabolic support and immune defenses [5,6]. Several studies have also verified the implication of EVs in the pathogenesis of neurodegenerative diseases (NDDs), confirming their involvement in, e.g., the diffusion of signals resulting in neuronal damage [7,8,9,10].

Cognitive impairment is one of the most common clinical features in NDs not necessarily related to aging. Given what is called “cognitive reserve”, the intellectual functions stay clinically unchanged until the disease enters an advanced stage—which however is also a negative prognostic factor since the first symptoms eventually emerge when the anatomical damage is advanced and almost irreversible [11,12]. One of the most challenging targets of medical research is to identify reliable biomarkers of early cognitive impairment to prevent its clinical onset and address more successful therapeutic efforts to counteract its progression. However, the published data report contradictory results, mostly influenced by the methods used for EVs extraction and the circulating compartment examined (i.e., plasma, serum, CSF).

In the last years, our research group has investigated EVs characterization and their RNA cargo [13,14], with particular interest in small RNAs such as microRNAs (miRNAs), in order to search for reliable and non-invasive methods for identifying circulating biomarkers associated with a given disease or suggestive of a peculiar phenotype [15]. However, several difficulties emerged, especially related to the very low yield of vesicular RNA that negatively impacted the use of extensive and innovative methods like the High Throughput Next Generation Sequencing (HT-NGS). To address both issues, we optimized vesicular RNA extraction from limited amounts of peripheral blood and adapted the highly sensitive high throughput OpenArray technology through specific modifications, resulting in improved miRNA expression data quality.

Based on these premises, here we present a pilot observation on a selected cohort of older subjects without and with early signs of cognitive impairment, in which the expression of 223 miRNAs (completed list in Supplementary Files), reported to be associated with brain disorders, neuroinflammation, and neurodegenerative diseases (NDs) was investigated in blood samples. The main purpose of the study was to search for candidate circulating biomarkers in EVs possibly associated with global cognitive abilities. By examining potential discrepancies between miRNA content and expression of EV-associated and non-vesicular plasma samples, we also aim to verify the most suitable strategy for identifying circulating biomarkers suggestive of cognitive deterioration.

## 2. Results

### 2.1. Clinical and Demographic Characteristics

A cohort of 43 subjects took part in this prospective molecular investigation (Table 1). Among them, 35 subjects were randomly included in the study among those admitted to the Center of Alzheimer Italy Association in Bari with possible age-related cognitive impairment at different stages. Twenty were females and 15 males, the mean age was 76.3 ± 6.8 years (range 67.8–88.5 years), and the mean years of schooling was 13.47 ± 3.93 years. The baseline evaluation of their cognitive status returned 23 cognitively preserved (CP) subjects (MMSE > 27) and 12 cognitively impaired (CI) (mean MMSE = 23.6 ± 2.5). Eight controls (Ctrl, 5 females, 3 males) were included in the evaluation, with MMSE = 30 and age range 69.2–78.6 years. In the overall study population, the MMSE score showed a significant—although moderate—correlation with age (r = −0.47, *p* < 0.05).

### 2.2. miRNA Expression in EV-Enriched Fractions

In our samples, the EV concentration range (by Nanoparticle Tracking Analysis, NTA) was 1.52 × 10^7^–1.37 × 10^8^ particles/mL. No significant difference resulted from the comparisons between the study groups (CI = 6.38 ± 2.7 × 10^7^; CP = 6.56 ± 4.1 × 10^7^; Ctrl = 7.25 ± 1.99 × 10^7^: *p* > 0.5). By analyzing the subjects according to their cognitive status, the Mann–Whitney U-test identified 30 candidate miRNAs out of 223 showing differential expression between the groups, with a *p*-value < 0.05 (Table 2). In CI subjects, the expression of all the miRNAs was significantly lower than that in the CP and Ctrl subgroups. Furthermore, hsa-miR-125a-5p and miR-223-3p seem to uniquely discriminate CI from Ctrl subjects (delta-Cq and *p*-value, respectively: −5.49 and 0.039; −8.17 and 0.048), whereas no miRNA was significantly different between CP and Ctrl subgroups. (See complete data in Supplementary Table S1a). A sensitivity analysis using *p*-values from multivariable linear models including age and sex as covariates confirmed the direction of effect for all identified miRNAs and supported the nominal *p*-value signals for 19 of them. Notably, miR-223-3p and miR-16-5p emerged as the most robust candidates, retaining an FDR ≤ 0.05 (completed data in Supplementary Table S1b). The same analysis was performed by sex, confirming the lower expression in CI females of miR-17-5p, miR-191-5p, and miR-199a-3p compared to CP females, and of miR-16-5p, miR-192-5p, and miR-223-3p in the corresponding male subgroups (*p* < 0.05) (in Supplementary Tables S2 and S3). For the explorative purpose of this analysis, we also performed a Spearman correlation analysis, with age and sex as confounding variables, to evaluate the association between vesicular miRNA expression across all 43 subjects and their MMSE score. This analysis was conducted to assess whether the observed miRNA alterations reflect not only group-level differences but also a continuous relationship with the degree of cognitive impairment. Data showed a positive correlation with MMSE in the expression of 15/30 of these miRNAs (miR-106b-5p, miR-128-3p, miR-15a-5p, miR-16-5p, miR-17-5p, miR-191-5p, miR-192-5p, miR-199a-3p, miR-221-3p, miR-222-3p, miR-223-3p, miR-22-3p, miR-26a-5p, miR-335-5p) (Supplementary Table S4).

### 2.3. miRNA Targets and Pathways Prediction

**Figure 1:**
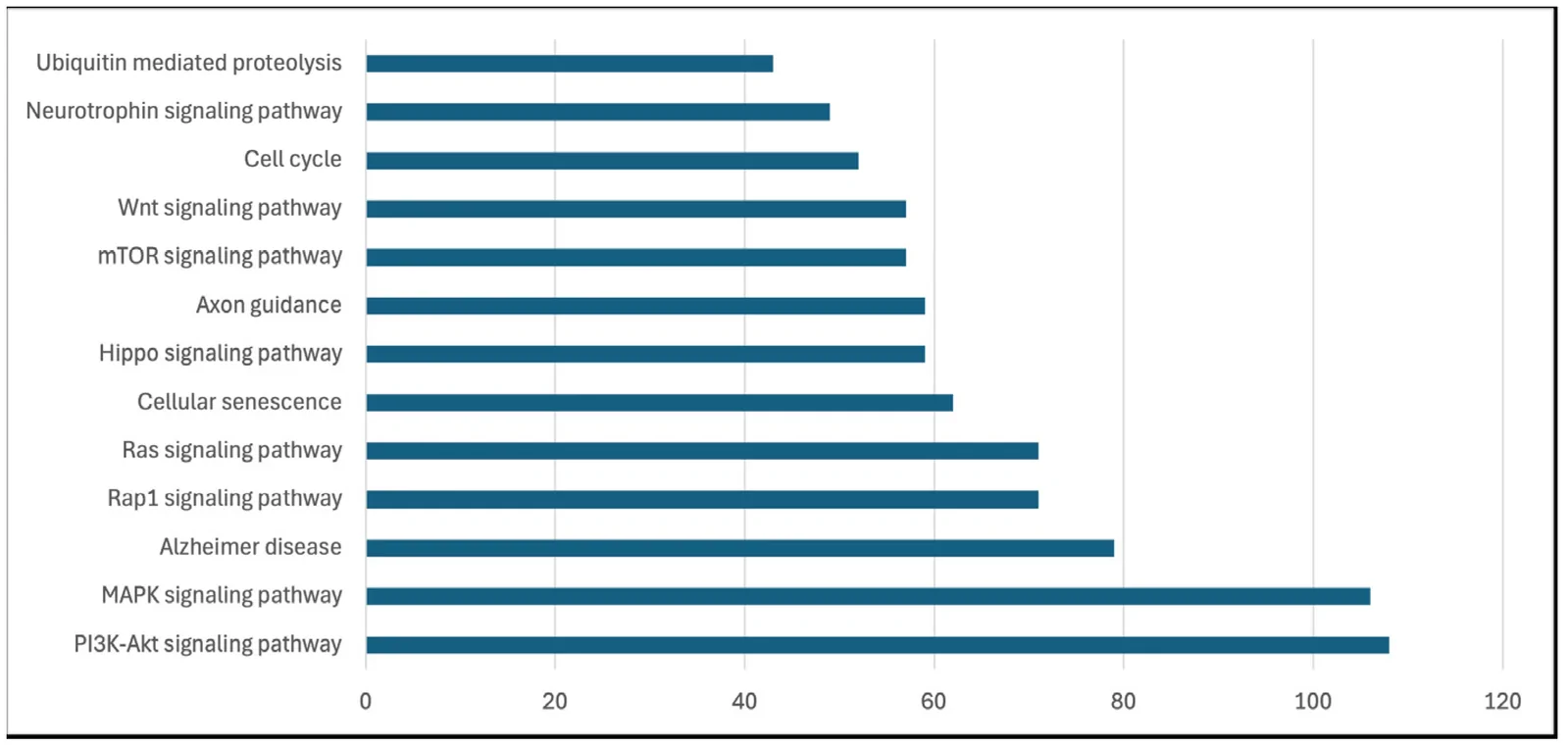
Representation of the pathway enrichment analysis associated with DE miRNAs in CI vs. CP (Section 2.3, selection by DAVID). The Table below summarizes (from left to right): the KEGG terms, the number of genes recruited for the *p*-value at Bonferroni test and the False Discovery Rate (FDR).

The search for genes targeted by the 30 miRNAs associated with cognitive deterioration in our study listed 6006 genes with very high scores (miRDIP). The enrichment analysis (DAVID) identified suggestive KEGG pathways already mentioned in NDs with cognitive involvement, e.g., PI3K-Akt signaling [16], Ras/MAPK signaling [17] Wnt signaling [18], the Cell-cycle pathway [19], and GOTerms such as GO:0046872 “metal ion binding” (1711 genes, *p* = 2.81 × 10^−27^), and GO:0008270 “zinc ion binding” (934 genes, *p* = 3.19 × 10^−15^). Figure 1 summarized the most suggestive networks (details in Supplementary Table S6).


**KEGG TERM**

**No. Genes**

**Bonferroni**

**FDR**
PI3K-Akt signaling pathway1080.0000003833.11 × 10^−08^MAPK signaling pathway1064.94 × 10^−12^1.06 × 10^−12^Alzheimer disease791.00 × 10^+00^0.1Rap1 signaling pathway710.000002361.18 × 10^−07^Ras signaling pathway710.0003770.00000582Cellular senescence621.44 × 10^−08^1.87 × 10^−09^Hippo signaling pathway590.0000004333.12 × 10^−08^Axon guidance590.0002710.00000429mTOR signaling pathway570.000004730.00000017Wnt signaling pathway570.0001980.00000357Cell cycle520.000570.0000086Neurotrophin signaling pathway490.0000005553.6 × 10^−08^Ubiquitin mediated proteolysis430.04440.000468

**Figure 2:**
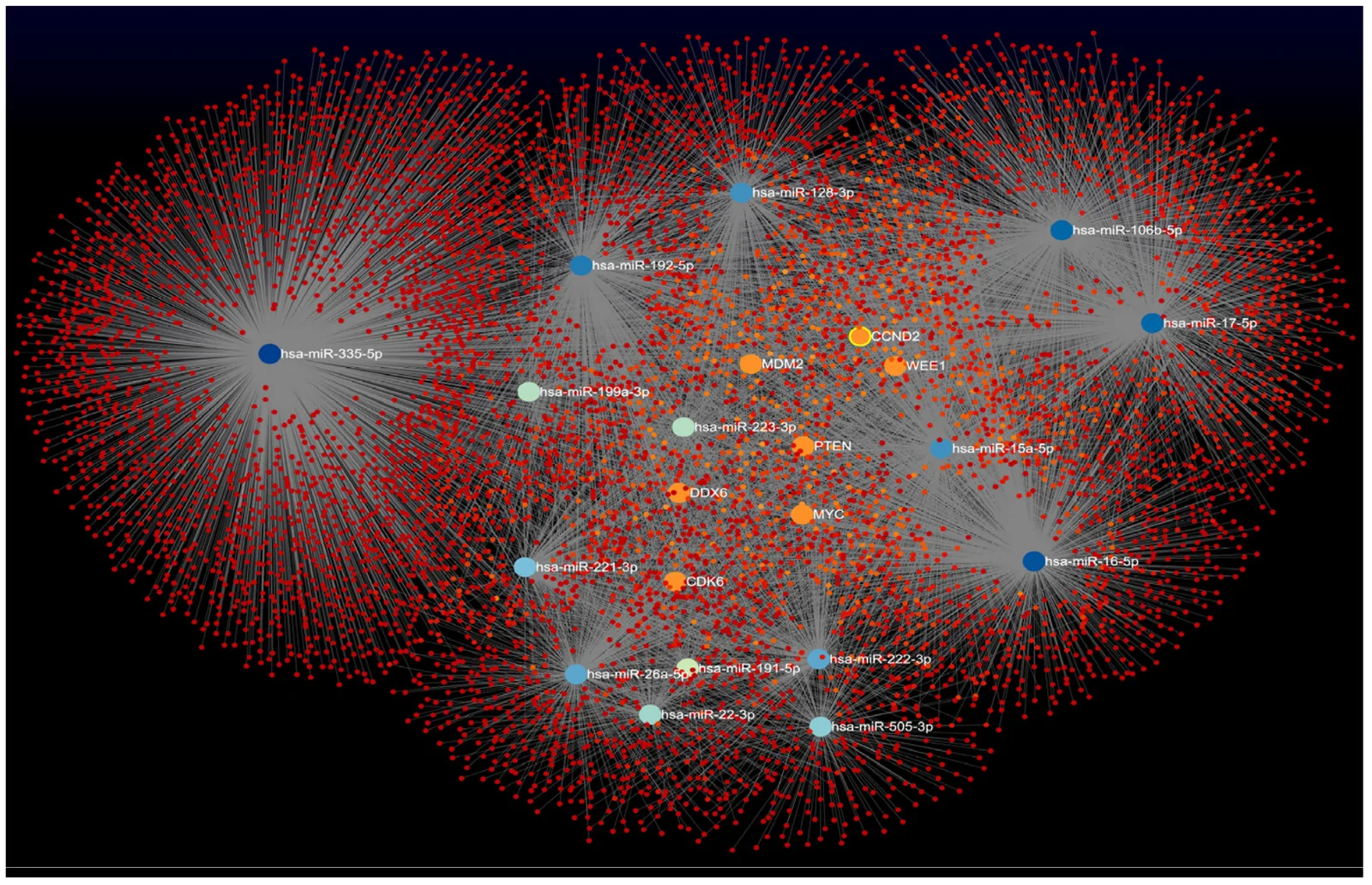
Network visualization (MiRNet) representing the interaction between the 15 miRNAs that seem to be associated with MMSE scores (both CI vs. CP comparison and Spearman correlation) and their predicted target genes. Genes like *MDM2*, *MYC*, *CCND2*, *DDX6*, *WEE1*, and CDK6 stand out as key interactions with multiple miRNAs, as well as for their functional role in cognitive abilities (see Section 3). Legend to Figure 2: miRNAs in dark-to-light blue (according to their degree of connections), target genes in red, shared genes in orange.

Of interest is that 15 miRNAs resulted from both the CI vs. CP comparison and the Spearman correlation. The interactions between their target genes (7118 and 11,048 edges) highlighted several genes that have already been identified as involved in cognitive functions and/or neurodegeneration such as *MDM2* [20], *MYC* [21], *CCND2* [22], *DDX6* [23], *WEE1* and *CDK6* [24] (Figure 2).

### 2.4. miRNA Expression in Pairs of EV-Associated and Unfractionated Plasma Samples

**Figure 3:**
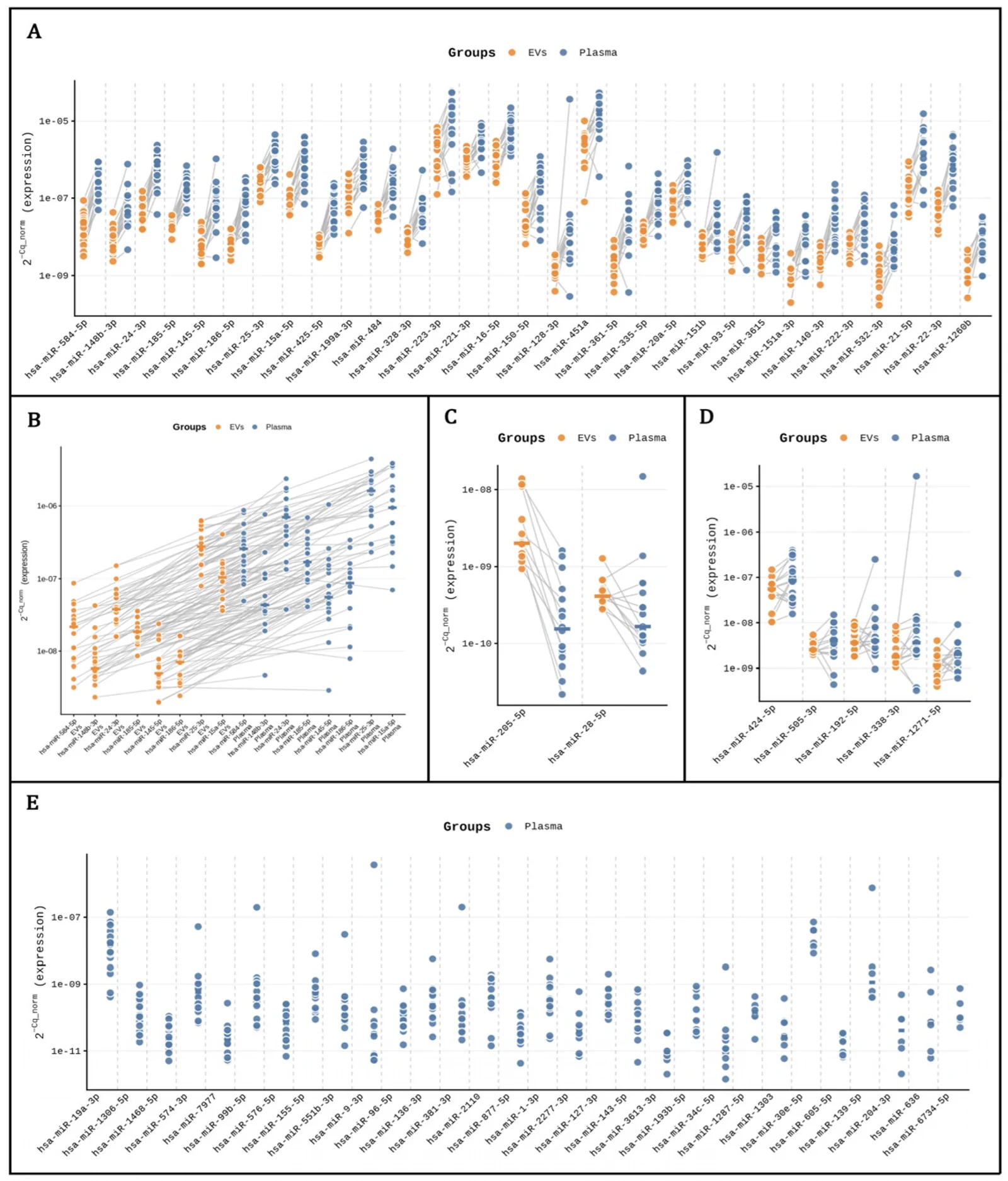
Graphic representation of the miRNA expression within EV and plasma pairs (in blue the plasma aliquots, in orange the EVs). (**A**) DE miRNA shared by at least 10 pairs and (**B**) 15 pairs, upregulated in plasma samples; (**C**) miRNAs upregulated in EVs compared to plasma; (**D**) miRNAs almost “stable” within the EV and plasma pairs; (**E**) miRNAs expressed uniquely in plasma samples (in X-axes: miRNA identifications; Y-axes = miRNA expression (2^−Cq_norm^).

Finally, to investigate potential discrepancies in miRNA quantification between EVs and plasma, the expression of the same 223 miRNAs was additionally assessed in plasma samples for a subset of 17 subjects (3 CI, 11 CP, and 3 controls). Paired EV-plasma data from these individuals showed that 72 out of 223 miRNAs were successfully amplified in both matrices, whereas 30 miRNAs were amplified only in plasma samples (Figure 3E). Figure 3 represents the different sets of results. By considering the miRNAs “shared” in at least 10 EV-plasma pairs, the analysis showed that 31 miRNAs were significantly (FDR < 0.05) upregulated in plasma aliquots compared to paired EV samples (Figure 3A), including eight miRNAs shared by at least 15 pairs (Figure 3B). Only two miRNAs displayed the opposite trend, although not uniformly across all subjects for hsa-miR-28-5p (Figure 3C). No miRNAs showed truly stable expression between the two sources, and when we explored whether any miRNAs exhibited limited variation between EVs and plasma, we identified a small group of five miRNAs whose expression levels were unchanged between EV and plasma pairs (Figure 3D). It is of interest that among the miRNAs associated with cognitive impairment in the previous analysis, 18 were upregulated in plasma compared to paired EVs, whereas two were stable in the two aliquots (details in Supplementary Table S7).

Taken together, in our view these observations support the notion that EV-associated and circulating plasma miRNAs represent complementary sources of information whose differential expression patterns may contribute to a more comprehensive characterization of cognitive impairment.

## 3. Discussion

This dataset collected from a cohort of 43 subjects older than 65 years showed that 30 miRNAs were identified as candidate miRNAs associated with the overall measure of cognitive abilities (MMSE score threshold) in EV-enriched fraction samples. Fifteen of them showed a direct correlation between expression and MMSE value. Since EV characterization was limited to NTA and no assessment of EV-associated or non-EV-associated markers was performed, the results should be interpreted as referring to plasma-derived EV-enriched fractions rather than to purified EVs. Although a contribution of co-isolated extracellular components cannot be excluded, the identified miRNA signature supports the value of EV-enriched plasma fractions as a promising source of candidate biomarkers of cognitive impairment in exploratory studies [25].

The identification of molecular signatures associated with age-related cognitive decline may represent a significant step toward the understanding of these progressive diseases, not to mention the valid support that this effort would offer to the selection of more targeted therapies. On the other hand, validated circulating biomarkers can be a valuable help in clinical practice, as they can contribute to the profiling of a more personalized medicine [26]. Non-coding RNAs (miRNAs, lncRNAs) seem to be major players in the orchestration of neurodegeneration [27], and many miRNAs carried by circulating EVs have been reported to be differentially expressed across several NDDs [28]. Among others, in AD, the expression of eight vesicular-associated miRNAs was analyzed in relation to additional instrumental parameters, including brain regional cortical thickness measured by MRI. Notably, some of these miRNAs were able to significantly discriminate among different stages of cognitive decline, as well as between AD and age-matched healthy controls [29].

Here we performed a cross-sectional investigation on a cohort of older subjects prospectively recruited at one of the AD Centers in Bari to possibly identify early signs of cognitive impairment suggestive of subsequent age-related dementias. Indeed, the subjects, examined at different stages of cognitive dysfunction, were submitted to a detailed neuropsychiatric battery that explored the main cognitive abilities according to international guidelines [30,31] (https://www.iss.it/-/snlg-diagnosi-e-trattamento-delle-demenze; https://www.nice.org.uk/guidance/ng97, accessed on 15 July 2024). However, given the exploratory nature of the study, we focused our analysis exclusively on the MMSE score, reserving other speculation to further in-depth analysis on larger populations.

In EV-enriched fractions, the downregulated expression of 30 miRNAs was associated with a poorer MMSE performance, which was consistent with the functional meaning of most of them (Table 2) and suggests that they may represent candidates for further investigation as circulating markers for use in longitudinal cognitive rehabilitation as well as in evaluating the disease progression. Among the others, we noticed that hsa-miR-223-3p was also downregulated in the comparison between CI and Ctrl, and it showed a decrease between Ctrl and CP (data available), which might confer to this miRNA the “proper” role as a candidate marker for monitoring the decline of cognitive abilities. Unfortunately, limitations, mainly due to the cohort size—especially in the subgroup of Ctrl—did not allow us to draw a conclusion on this issue. However, decline of miR-223-3p expression has been reported in animal models of anxiety/depression as it affects the NLPR3-inflammasome pathway, leading to cellular damage, impaired neural plasticity, and the suppression of neurogenesis [32]. Interestingly, Mancuso et al. [33] found a lower serum concentration of miR-223-3p to be associated with the severity of cognitive dysfunctions from Mild Cognitive Impairment (MCI) to AD compared to normal controls, confirming our findings and the attention to this miRNA as a potential circulating marker for age-related cognitive dysfunctions.

On the other hand, in silico experiments between the candidate miRNAs and their target genes seem to implicate suggestive genes that have been already identified as involved in cognitive functions, such as *MDM2* [20], *MYC* [21], *CCND2* [22], *DDX6* [23], *WEE1* [34] and *CDK6* [24]. Taken together, they evoked pathways already associated with ND, like PI3K-Akt signaling [16], Ras/MAPK signaling [17], Wnt signaling [18], and the Cell-cycle pathway [19], thus suggesting the possibility that this molecular process may impact, at least partially, the pathogenesis of the cognitive decline that we observed in our study population (Figure 2).

Other interesting findings from this investigation support the involvement of the metal ion binding network in age-related cognitive impairment, since 18/30 EV-associated miRNAs (miR-15a-5p, miR-16-5p, miR-17-5p, miR-21-5p, miR-22-3p, miR-24-3p, miR-26a-5p, miR-199a-3p, miR-7-5p, miR-221-3p, miR-30b-5p, miR-125b-5p, miR-128-3p, miR-130a-3p, miR-191-5p, miR-106b-5p, miR-361-5p, and miR-92b-3p) and their targeted genes resulted significantly engaged in this process. Published data reported that one of the mechanisms involved in dementias like AD may be the neurotoxicity caused by excessive metal accumulation in the CNS with inductions of aggregates that precipitate causing neuronal cell death [35,36]. The levels of heavy metals within CNS seem to be associated with the deregulation of several miRNAs related to AD [37,38]. Many miRNAs carried by EVs are differentially expressed during aging and are being increasingly recognized as aging biomarkers [28]. It is reasonable to believe that both these factors—metals and miRNAs—may be connected and possibly influence each other in the “EV environment” implicated in the cognitive decline of older subjects, as observed in Parkinson’s disease [39].

An additional purpose of the study was to verify that the molecular content of two aliquots from the same subject (plasma and total EVs) is not equivalent (in type or expression) and that the search for significant biomarkers should consider this discrepancy other than the methods of EVs isolation and the RNA extraction. On this ground, in our view this study deserves attention as it contains some peculiarities compared to other published reports. Above all, while other quite concomitant studies following similar strategies (for the molecular methods) investigated only one compartment—plasmatic EVs in AD [40], in Frontotemporal Dementia (FTD) vs. Primary Psychiatric Disorders (PPD) [41], in FTD vs. Bipolar Disorder (BD) [42]; and EVs extracted from CSF in AD subjects [43]—we started with plasmatic EVs but also performed some evaluation in paired EV and plasma samples. As far as we know, this is the first report exploring the paired expression of miRNAs in two concomitant compartments of elder subjects. Given the functional crosstalk between them, consistent results are expected, but the possibility that some “gradient” changes may occur in pathological conditions suggests caution in the comparison of significant miRNAs from different investigations. It was also intriguing to find that five miRNAs (miR-424-5p, miR-505-3p, miR-192-5p, miR-338-3p, miR-1271-5p) were almost stable between the pairs, as well as the expression of miR-205-5p and miR-28-5p (not in all pairs) were found upregulated in total EV-enriched fractions compared to plasma. Further confirmations are needed in this direction, e.g., the full characterization of EVs cargo and origin, before reaching more definitive conclusions.

As we mentioned earlier, a limitation of the present study is the level of EVs characterization, which was limited to the evaluation of particle size distribution and concentration assessed by NTA. However, the primary objective was to investigate miRNA signatures associated with plasma-derived EV-enriched fractions, with a focus on their potential biological/clinical relevance. Therefore, we believe that our approach was consistent with some literature in which EV preparations are used as a source of circulating regulatory RNAs rather than being characterized as fully defined vesicle populations [44,45]. Therefore, throughout the manuscript, we adopted the more conservative term “EV-enriched fraction” rather than “purified EVs”. Consequently, the observed miRNA signatures should be interpreted as associated with plasma-derived EV-enriched fractions, acknowledging the potential contribution of co-isolated extracellular particles and plasma components.

Further limitations were mostly due to the sample size. (See Section 4). This was noticed mainly for the comparison with the Ctrl group (n.8 subjects) that did not allow proper confrontation to find any significant molecular discrepancies, e.g., with CP. In addition, in the female Ctrl subgroup the analysis showed a smaller number of expressed miRNAs, but we cannot determine whether this was a sampling or sex bias. The prospective design of the study, for which all subjects were submitted to the neuropsychological evaluation after the recruitment, could not create a more consistent group of CI subjects. Finally, a plasma sample contemporary to total EV fraction was available only for 17 subjects due to random technical errors in the blood collection, so no selection bias should be declared.

## 4. Materials and Methods

### 4.1. Study Participants

Older subjects (age > 65 years) were recruited at the Neuropsychology service of the Alzheimer Italy Association in Bari. They were preliminarily investigated for secondary causes for dementia by neurologists with focused experience in age-related diseases. Demographic and clinical information were collected at the time of their study entry. All subjects or their legal guardians signed written informed consent forms (according to the Declaration of Helsinki) at the time of the enrollment. The study was approved by the local Ethic Committee of IRCCS Istituto Oncologico “Gabriella Serio”, Bari, Italy (protocol number: 388/2023).

For the purpose of the study, the Mini Mental State Examination (MMSE) test [46] was given to the recruited subjects by trained psychologists with focused experience in the evaluation of the older adults, returning a final score that was adjusted according to age and education [47]. After a detailed anamnestic, clinical and psychological interview, and evaluation of neurological reports, the Activities of Daily Living (ADL) [48] and Instrumental ADL (IADL) [49] geriatric rating scales were applied to the participants and their relatives to measure the degree of self-sufficiency and functional autonomy of individuals with regard to basic personal care activities and more complex activities necessary for living independently in the community. This procedure allows a better diagnostic evaluation for discriminating between mild and major cognitive disorder as reported by DSM-5 diagnostic criteria [50]. Subjects with MMSE score >27 were considered cognitively preserved (CP) and distinguished from mild to severe cognitive impairment (CI) [51].

Age-related control subjects (Ctrl), negative for any neurodegenerative disorders and with an MMSE = 30 were also included in the study. They were selected among non-consanguineous family members (i.e., spouses) or staff members and volunteers of AI.BA.

### 4.2. Sample Collection

Peripheral blood samples (6 mL) were collected from all the consenting subjects in vacutainer tubes containing EDTA (ethylenediaminetetraacetic acid), which acts as an anticoagulant and binds calcium ions, disrupting the coagulation cascade. The tubes were stabilized at room temperature for approximately 20 min prior to plasma separation to minimize cold-induced platelet activation and artifactual release of extracellular vesicles and circulating RNA. The samples were then centrifuged at 1500 *g* for 15 min [52] to obtain plasma. All these steps are crucial to maintain the plasma’s stability and aid the miRNA recovery. The obtained plasma was split into aliquots of 500 µL/each to prevent RNA degradation and changes in miRNA expression patterns during the frozen/unfrozen procedure. When it was not possible to immediately proceed to the next steps, the aliquots were stored at −20 °C for just a few hours, or at −80 °C for long-term storage [52].

The plasma samples belonging to the whole study group were submitted to EVs isolation followed by miRNAs extraction (see below for details). For 17 randomly selected subjects, we examined the miRNA’s expression both in the plasma and in the plasmatic-EV fraction, to verify the individual difference—if any—between the two matrices. Both the aliquots were submitted to a further 1500 *g* centrifugation for 15 min to remove all the debris before the subsequent processing (especially in cases of previous freezing).

### 4.3. EVs Isolation (EV-Enriched Fractions)

Total EVs were extracted from plasma samples using an innovative column based on size exclusion chromatography, SmartSEC (System Biosciences, Newark, CA, USA, CAT SSEC200A-1), using a single-tube format to extract vesicles in a range from about 40 to 120 nm, likely attributable to exosomes. Briefly, after stabilizing the column at RT (for at least 30 min), it was centrifuged at 500 *g* for 30 s to remove the storage buffer, then washed with a provided column buffer and centrifuged again to stabilize the beads.

**Figure 4:**
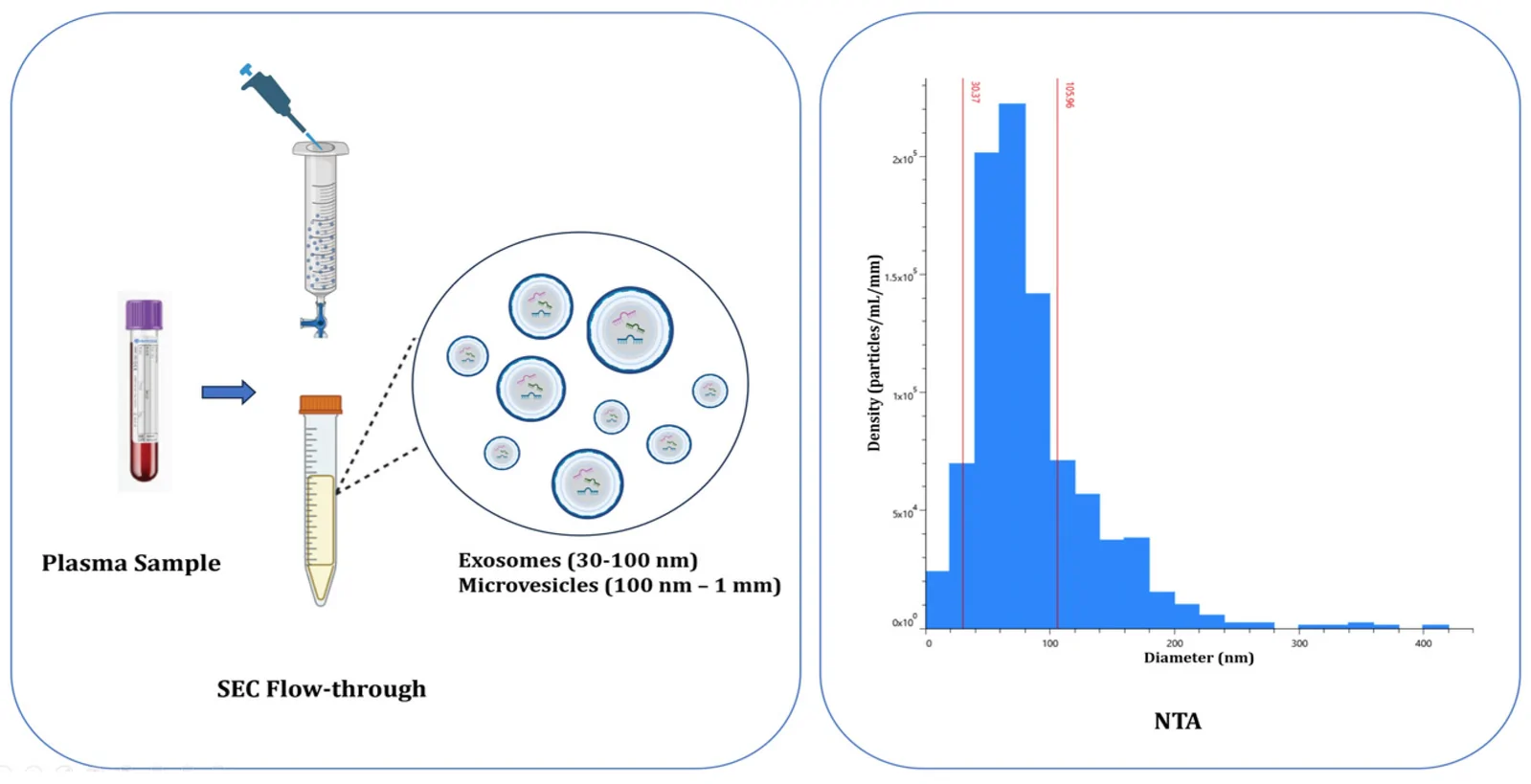
Summarized workflow for EV isolation: from plasma sampling to SEC processing, then to NTA measurement.

Since the protocol is optimized for 100 µL up to 250 µL of plasma, we used the maximum plasma volume to obtain the maximum RNA yield. Each aliquot of plasma was first centrifuged at 10,000–12,000 *g* for 15 min to remove cellular debris and large vesicles, then 250 µL was added on top of the column with an equal volume of fresh PBS to bring the total volume up to 500 µL and incubated at RT for 30 min with a medium speed rotation. The column was placed in a clean collection tube and centrifuged at 500 *g* for 30 s to collect the EVs (Figure 4).

The size distribution and concentrations of isolated EVs were verified by a multi-laser nanoparticle tracking analysis (NTA) instrument (ViewSizer 3000, HORIBA-Irvine, CA, USA), an integrated platform that provides statistically robust size distributions, direct quantification of particle concentration, and assessment of the population heterogeneity of EVs (https://www.horiba.com/int/scientific/products/detail/action/show/Product/viewsizer-3000-1-1862/) (access date 24 October 2025).

Although we were aware that MISEV2023 [53] recommends the use of complementary characterization approaches, including the assessment of EV-associated and non-EV-associated markers, for the scope of this study and the small amount of plasma aliquots, we limited this step to NTA analysis. On this ground, we refer to our samples as ‘EV-enriched fractions’ rather than definitive EV preparations. This terminology was chosen to reflect the level of characterization performed and to avoid overinterpretation of the particle identity. NTA was used to demonstrate the presence of nanoparticles within the expected EV size range; no claim of purification or exclusive vesicular composition is made. Therefore, the contribution of non-vesicular components cannot be excluded [54], and the identified miRNA profile should be interpreted in the context of plasma-derived EV-enriched fractions

### 4.4. miRNA Isolation (From Plasma and EV-Enriched Aliquots)

The miRNA extraction was performed from a sample input of 100 µL (plasma/EVs) using the miRNeasy serum/plasma kit (QIAGEN-Hilden, Germany) following the total RNA including miRNA isolation procedure. A spike-in exogenous control (ath-mir-159, 1 µL, 10 pM) was added to each sample. The yield and quality of the obtained RNAs were analyzed using a Qubit Fluorometer (ThermoFisher Scientific, Waltham, MA, USA) and an Agilent 2100 Bioanalyzer (Agilent Technologies, Santa Clara, CA, USA), respectively, then the extracted RNA was stored at −80 °C until use.

The mean total RNA concentration was 6.9 ± 7.4 ng/mL (in EVs) and 12 ± 2.8 ng/mL (in the plasma samples), respectively.

### 4.5. Workflow for Preparing the OpenArray Plates

A high processivity and sensitive Open Array technology (by QuantStudio 12K-Flex, ThermoFisher, USA) was used. Since few published protocols were available, especially for low RNA inputs [40,43], we made use of this experience to develop some methodological suggestions to be applied to further studies.

Highly sensitive-specific cDNA synthesis was obtained from miRNA molecules by TaqMan Advanced miRNA Assays (Life Technologies, ThermoFisher Scientific, Waltham, MA, USA). The protocol provides a first reaction with a sample input of 2 µL performed with a polyadenylation at 37 °C for 45 min, turned at 65 °C for 10 min to stop the reaction. The second step is the ligation of an adapter sequence to extend the mature miRNA, performed at 16 °C for 1 h. Reverse transcription was performed at 42 °C for 15 min, turned at 85 °C for 5 min to stop the reaction. To proceed with the amplification, the reaction was performed with enzyme activation at 95 °C for 5 min, 22 cycles of denaturation at 95 °C for 3 s in addition to annealing and extension at 60 °C for 30 s, followed by 10 min at 99 °C for stopping, with the maximum ramp speed.

The workflow continues with the cDNA dilution in a ratio of 1:20 and the addition of the TaqMan OpenArray Real Time Master MIX (Life Technologies, ThermoFisher Scientific, Waltham, MA, USA). Considering the low concentration of the starting RNA, we performed this step without the cDNA dilution suggested by the provider to obtain the maximum amplification. According to the protocol, a mixed volume requested for the 224 targets reaction was loaded in a 96-well plate. It was composed of 12.5 µL of not-diluted cDNA to a corresponding volume of TaqMan OpenArray Real Time Master MIX [55]. Since an array consists of 3072 holes, the OpenArray system requires the use of a robotic sample loader (OpenArray Acufill System, Life Technologies, ThermoFisher Scientific, Waltham, MA, USA) that automatically transfers the mix from a 384-well plate to the OpenArray plate to complete the preparation set-up and start the runs.

### 4.6. Statistics

Given the extremely low expression levels of the molecules under investigation, particular attention was dedicated to defining an appropriate statistical framework, and non-parametric statistical tests were adopted as the most suitable analytical approach. Raw Cq values were first filtered with the following thresholds: Cq ≥ 36, amplification score ≥ 1, and Cq confidence ≥ 0.8. At this stage, careful consideration was also given to the choice of data-normalization strategy, as this step has a critical influence on the downstream statistical analyses. An exploratory evaluation of data distribution through histograms and scatter plots revealed that the “global normalization” approach proposed by ThermoFisher was insufficient to correct scale deviations observed in our dataset. For this reason, normalized Cq values (Cq_norm) were computed by subtracting sample-specific size factors, defined as the median difference between each target’s Cq and its target-wide mean. Expression values used for visualization were subsequently derived as 2^-Cq_norm.

Group comparisons of miRNA expression (CI vs. CP, CI vs. Ctrl, CP vs. Ctrl, and sex-stratified analyses) were performed using the Mann–Whitney U-test. Since the study focused on very low-expressed targets, and to appropriately encode the absence of expression attributable to Cq values above threshold or undetermined measurements, a placeholder value of 40 (right-censoring) was assigned to all null-expression observations (any value above 36 produces the same effect within a rank-based test). For the comparison between EV-enriched fractions and plasma, a paired Wilcoxon signed-rank test was applied, and ΔCq_norm values were calculated for each miRNA with a determined expression value in both matrices.

As the study represents a qPCR-based validation performed on a QuantStudio platform, the selection of statistical outcomes relied on nominal *p*-values, considered as an initial screening criterion to prioritize candidate miRNAs for further investigation. For transparency and enhanced interpretability, we also report FDR-adjusted *p*-values using the Benjamini–Hochberg procedure, although these were not used as decision criteria. Results were considered worthy of further investigation if *p*-values ≤ 0.05 and absolute ΔCq_norm ≥ 1.

Even though the use of non-parametric tests was preferred for primary analyses due to the presence of right-censored values, which are not appropriately handled by standard linear modeling approaches, we performed a sensitivity analysis using *p*-values from multivariable linear models including age and sex as covariates (limma framework). The results should be interpreted with caution and considered only as a support for the overall biological trend observed. FDR values are reported in Supplementary Tables.

To assess potential associations between miRNA expression levels and MMSE scores across all participants (CI, CP, and Ctrl), Spearman correlation analyses were conducted, adjusted by sex and age as covariates. Correlation results were selected when the absolute value of rho exceeded 0.3 and the corresponding *p*-value was below 0.05.

Data analyses were conducted in R (version 4.5.2; R Core Team), using the packages modeest (v2.4.0), limma (v1.1), and ppcor (v3.68.0 from Bioconductor v3.23).

To check for any common functional pathway possibly recruited by the miRNAs of interest and their computationally predicted target genes, the web-based software mirDIP (https://ophid.utoronto.ca/mirDIP/) (Version 5.3.0.3, Database version 5.2.2.1, access date: 15 November 2025), TarBase v9.0 (https://dianalab.e-ce.uth.gr/tarbasev9), DAVID (https://davidbioinformatics.nih.gov/) access date: 15 May 2026, MiRNet v.2.0 (https://www.mirnet.ca/miRNet/home.xhtml) access date: 15 October 2025, and miRPath v4.0 (http://62.217.122.229:3838/app/miRPathv4) access date: 15 December 2025 were used.

## 5. Conclusions

Being able to intercept the very early signs of cognitive decline still represents a big challenge for the healthcare of a given Country, with both social and economic consequences. On the other hand, disentangling the molecular networks implicated in early cognitive deterioration may represent a valid support in the identification of novel therapies.

Overall, our findings provide preliminary evidence that specific EV-associated miRNAs may serve as candidate biomarkers of cognitive impairment in aging subjects. Nevertheless, the statistical power of the study is limited by the sample size, and independent validation in larger and well-characterized cohorts will be necessary to confirm these associations and assess their potential translational applicability.

## Figures and Tables

**Table 1:** Study population. Descriptive demographic and clinical features of the enrolled subjects. Data are presented in the whole study population (No. 43) by their cognitive status, evaluated by MMSE score.

	Study Population (No. = 43)	CP (No. = 23)	CI (No. = 12)	Ctrl (No. = 8)
Female/Male	25/18	12/11	8/4	5/3
Age at sampling (years)	75.15 ± 6.8	74.63 ± 5.95	78.59 ± 8.11	71.46 ± 5.45
Schooling (years)	13.81 ± 3.84	13.86 ± 3.93	11.75 ± 4.71	13.24 ± 4.01
MMSE score	28.18 ± 3.04	29.72 ± 0.77	23.55 ± 2.53	30

*Legend*: CP = cognitively preserved, CI = cognitively impaired; Ctrl = controls; MMSE = Mini Mental Scale examination.

**Table 2:** miRNAs possibly associated with cognitive impairment. Below the list of 30 candidate miRNA downregulated in CI compared to CP subjects. (Data represented by *p*-value, ΔCq_norm_, False Discovery Rate and Sensitivity, see Statistics for details of the analysis). The last two columns report the published citations in which the same miRNA were significantly associated with AD or other disorders with cognitive implications and the comparison with our data (C = concordant; NC = not concordant), respectively.

	CI *Versus* CP					
miRNA ID	*p*-Value	ΔCq_norm_	FDR	Sensitivity	Citations (*)	Comparison
hsa-miR-125b-5p	0.0428	−3.59	0.1590	0.1060	AD	C/NC
hsa-miR-223-3p	0.0032	−8.19	0.0837	0.0004	Anxiety and depression	C
hsa-miR-15a-5p	0.0030	−7.78	0.0837	0.0060	Aging and cognitive decline	C
hsa-miR-409-3p	0.0127	−4.32	0.1066	0.0444	Aging stem cells	C
hsa-miR-22-3p	0.0053	−7.62	0.0925	0.0277	AD	C
hsa-miR-505-3p	0.0133	−5.32	0.1066	0.0657	Aging	NC
hsa-miR-106b-5p	0.0428	−4.80	0.1590	0.0419	AD	NC
hsa-miR-30b-5p	0.0474	−4.39	0.1643	0.0797	MS	C
hsa-miR-16-5p	0.0032	−6.80	0.0837	0.0005	AD	C
hsa-miR-130a-3p	0.0455	−4.05	0.1630	0.1475	AD	C
hsa-miR-17-5p	0.0032	−5.70	0.0837	0.0184	AD	NC
hsa-miR-335-5p	0.0077	−7.19	0.1066	0.0274	AD	C
hsa-miR-92b-3p	0.0276	−6.75	0.1282	0.0347	Cognitive domains	C
hsa-miR-26a-5p	0.0154	−5.70	0.1066	0.0249	AD	C
hsa-miR-192-5p	0.0097	−6.21	0.1066	0.0307	Cognition in PD	C
hsa-miR-222-3p	0.0135	−6.48	0.1066	0.0196	Cognitive functions in musicians	NC
hsa-miR-221-3p	0.0284	−7.78	0.1282	0.0398	Neuronal apoptosis	C
hsa-miR-7-5p	0.0340	−5.34	0.1424	0.0587	AD	C
hsa-miR-328-3p	0.0342	−5.56	0.1424	0.0897	MDD, ADHD	NC
hsa-miR-532-3p	0.0196	−4.39	0.1239	0.0577	Experimental cognition	C
hsa-miR-128-3p	0.0229	−5.58	0.1252	0.0203	AD	C
hsa-miR-199a-3p	0.0042	−7.77	0.0879	0.0038	AD	C
hsa-miR-361-5p	0.0282	−4.97	0.1282	0.1246	Music listening	C
hsa-miR-21-5p	0.0241	−5.11	0.1252	0.1028	AD	NC
hsa-miR-140-3p	0.0203	−5.44	0.1239	0.0622	AD	C
hsa-miR-24-3p	0.0216	−7.34	0.1248	0.0051	AD	C
hsa-miR-152-3p	0.0407	−3.85	0.1590	0.2658	AD	C
hsa-miR-584-5p	0.0083	−6.31	0.1066	0.0150	Stroke	NC
hsa-miR-191-5p	0.0128	−5.45	0.1066	0.0089	AD	C
hsa-miR-151a-3p	0.0153	−5.58	0.1066	0.0159	AD	C

(*) Details of the published studies are in Supplementary Table S5. Legend: AD = Alzheimer’s Disease; PD = Parkinson’s Disease; MDD = Major Depressive Disorder; ADHD = Attention Deficit/Hyperactivity Disorder.

## Data Availability

The Authors are going to upload the raw molecular data in a public repository (details will be soon available).

## Supplementary Materials

The following supporting information can be downloaded at: https://www.mdpi.com/article/10.3390/ijms27177838/s1.

## Abbreviations

The following abbreviations are used in this manuscript:

EV	Extracellular Vesicles
ND	Neurodegenerative Diseases
AD	Alzheimer’s Diseases
MMSE	Mini Mental State Examination
CP	Cognitively preserved
CI	Cognitively Impaired
miRNA	microRNA
FDR	False Discovery Rate
NTA	nanoparticle tracking analysis
SEC	size exclusion chromatography
HT-NGS	High Throughput Next Generation Sequencing
