# Supplementary material for: Low-Sample Input miRNA Profiling in Extracellular Vesicles-Enriched Plasma Fractions from Older Adults With/out Cognitive Impairment
Source: Int J Mol Sci. 2026 Sep 1;27(17):7838. doi: 10.3390/ijms27177838 (PMC13565787; doi:10.3390/ijms27177838)
Supplement: Supplementary file 1 [file ijms-27-07838-s001.zip › REVISED_SupplTables.pdf]

Supplementary Table S1a:

Below the list of miRNAs resulted significantly dysregulated in CI vs. CP vs. Ctrl subjects (each set of data has been represented by p-value, FDR and ΔCq_{norm})..

	CI vs CP			CI vs Ctrl			CP vs Ctrl		
miRNA	p-value	FDR	ΔCq_{norm}	p-value	FDR	ΔCq_{norm}	p-value	FDR	ΔCq_{norm}
hsa-miR-125b-5p	0.0428	0.1590	-3.59	0.0396	0.6346	-5.49	0.3285	0.9371	-1.90
hsa-miR-223-3p	0.0032	0.0837	-8.19	0.0486	0.6346	-8.17	0.8505	0.9533	0.02
hsa-miR-15a-5p	0.0030	0.0837	-7.78	0.0540	0.6346	-6.87	0.7262	0.9371	0.91
hsa-miR-409-3p	0.0127	0.1066	-4.32	0.0596	0.6346	-3.32	0.6087	0.9371	1.00
hsa-miR-22-3p	0.0053	0.0925	-7.62	0.0634	0.6346	-6.86	0.9350	0.9637	0.76
hsa-miR-505-3p	0.0133	0.1066	-5.32	0.0683	0.6346	-4.73	0.8434	0.9533	0.59
hsa-miR-106b-5p	0.0428	0.1590	-4.80	0.0683	0.6346	-5.38	0.6636	0.9371	-0.58
hsa-miR-30b-5p	0.0474	0.1643	-4.39	0.0914	0.6346	-4.60	1	1	-0.21
hsa-miR-16-5p	0.0032	0.0837	-6.80	0.0964	0.6346	-6.22	0.7671	0.9427	0.58
hsa-miR-130a-3p	0.0455	0.1630	-4.05	0.1050	0.6346	-4.02	0.8639	0.9578	0.03
hsa-miR-17-5p	0.0032	0.0837	-5.70	0.1695	0.6346	-4.02	0.7062	0.9371	1.67
hsa-miR-335-5p	0.0077	0.1066	-7.19	0.1701	0.6346	-4.40	0.2483	0.9371	2.79
hsa-miR-92b-3p	0.0276	0.1282	-6.75	0.1701	0.6346	-4.83	0.9338	0.9637	1.92
hsa-miR-26a-5p	0.0154	0.1066	-5.70	0.1847	0.6346	-3.64	0.5702	0.9371	2.07
hsa-miR-192-5p	0.0097	0.1066	-6.21	0.2124	0.6346	-3.88	0.5454	0.9371	2.33
hsa-miR-222-3p	0.0135	0.1066	-6.48	0.2617	0.6666	-3.67	0.1870	0.9371	2.81
hsa-miR-221-3p	0.0284	0.1282	-7.78	0.2652	0.6666	-4.40	0.8502	0.9533	3.37
hsa-miR-7-5p	0.0340	0.1424	-5.34	0.3184	0.7627	-2.95	0.5454	0.9371	2.39
hsa-miR-328-3p	0.0342	0.1424	-5.56	0.3778	0.7627	-3.11	0.6601	0.9371	2.44
hsa-miR-532-3p	0.0196	0.1239	-4.39	0.3801	0.7627	-2.10	0.5163	0.9371	2.29
hsa-miR-128-3p	0.0229	0.1252	-5.58	0.3827	0.7627	-2.84	0.0883	0.9371	2.74
hsa-miR-199a-3p	0.0042	0.0879	-7.77	0.4348	0.7627	-2.42	0.1534	0.9371	5.35
hsa-miR-361-5p	0.0282	0.1282	-4.97	0.4446	0.7627	-2.40	0.2483	0.9371	2.56
hsa-miR-21-5p	0.0241	0.1252	-5.11	0.4576	0.7627	-1.08	0.6244	0.9371	4.03
hsa-miR-140-3p	0.0203	0.1239	-5.44	0.5434	0.7627	-1.71	0.2299	0.9371	3.73
hsa-miR-24-3p	0.0216	0.1248	-7.34	0.5495	0.7627	-1.43	0.4999	0.9371	5.91
hsa-miR-152-3p	0.0407	0.1590	-3.85	0.6365	0.8000	-0.83	0.2761	0.9371	3.03
hsa-miR-584-5p	0.0083	0.1066	-6.31	0.8703	0.9627	-0.49	0.0584	0.9371	5.82
hsa-miR-191-5p	0.0128	0.1066	-5.45	0.8703	0.9627	-0.82	0.1597	0.9371	4.64
hsa-miR-151a-3p	0.0153	0.1066	-5.58	0.9503	0.9699	-0.58	0.0593	0.9371	5.00

Supplementary Table S1b:

Sensitivity analysis using multivariable linear models (limma) including age and sex as covariates. Bold results are selected by sensitivity analysis, italic results were selected by non-parametric test values presented in Supplementary Table 1a. The direction of effect is confirmed for all identified miRNAs, the nominal p-value signals supports 19 of them, miR-223-3p and miR-16-5p emerged with FDR ≤ 0.05 in CI vs CP comparison.

	CI vs CP			Ctrl vs CI			Ctrl vs CP		
miRNA	logFC	P-value	FDR	logFC	P-value	FDR	logFC	P-value	FDR
hsa-miR-223-3p	**-8.11**	**0**	**0.035**	**8.06**	**0.010**	0.823	-0.04	0.985	1
hsa-miR-125b-5p	-3.11	0.106	0.416	4.68	0.092	1	1.57	0.483	1
hsa-miR-16-5p	**-7.00**	**0**	**0.035**	**6.61**	**0.016**	0.823	-0.40	0.854	1
hsa-miR-199a-3p	**-7.54**	**0.004**	0.152	2.07	0.560	1	-5.48	0.065	1
hsa-miR-24-3p	**-7.25**	**0.005**	0.152	1.31	0.710	1	**-5.94**	**0.046**	0.911
hsa-miR-15a-5p	**-7.11**	**0.006**	0.152	5.72	0.112	1	-1.39	0.631	1
hsa-miR-191-5p	**-5.91**	**0.009**	0.194	1.64	0.597	1	-4.27	0.100	1
hsa-miR-584-5p	**-6.29**	**0.015**	0.221	0.48	0.893	1	-5.81	0.054	0.911
hsa-miR-151a-3p	**-4.76**	**0.016**	0.221	-0.81	0.768	1	**-5.56**	**0.017**	0.911
hsa-miR-17-5p	**-5.59**	**0.018**	0.222	3.84	0.247	1	-1.75	0.518	1
hsa-miR-222-3p	**-5.14**	**0.020**	0.222	1.42	0.643	1	-3.72	0.144	1
hsa-miR-128-3p	**-4.51**	**0.020**	0.222	1.04	0.699	1	-3.46	0.123	1
hsa-miR-26a-5p	**-5.34**	**0.025**	0.235	3.08	0.355	1	-2.26	0.408	1
hsa-miR-335-5p	**-5.59**	**0.027**	0.235	1.70	0.630	1	-3.88	0.185	1
hsa-miR-22-3p	**-6.48**	**0.028**	0.235	4.91	0.236	1	-1.57	0.641	1
hsa-miR-192-5p	**-4.91**	**0.031**	0.247	1.67	0.598	1	-3.23	0.218	1
hsa-miR-92b-3p	**-6.03**	**0.035**	0.265	3.64	0.364	1	-2.39	0.467	1
hsa-miR-221-3p	**-6.64**	**0.040**	0.290	2.52	0.578	1	-4.12	0.271	1
hsa-miR-106b-5p	**-5.12**	**0.042**	0.292	5.92	0.099	1	0.81	0.781	1
hsa-miR-409-3p	**-3.44**	**0.044**	0.293	1.84	0.444	1	-1.60	0.419	1
hsa-miR-532-3p	-3.54	0.058	0.321	0.68	0.794	1	-2.85	0.190	1
hsa-miR-7-5p	-3.79	0.059	0.321	0.34	0.903	1	-3.45	0.142	1
hsa-miR-140-3p	-3.56	0.062	0.325	-1.47	0.585	1	**-5.03**	**0.028**	0.911
hsa-miR-505-3p	-3.71	0.066	0.325	1.99	0.484	1	-1.71	0.463	1
hsa-miR-30b-5p	-3.74	0.080	0.370	3.53	0.246	1	-0.21	0.931	1
hsa-miR-328-3p	-4.43	0.090	0.371	1.20	0.744	1	-3.23	0.290	1
hsa-miR-21-5p	-5.18	0.103	0.414	1.23	0.784	1	-3.95	0.288	1
hsa-miR-361-5p	-3.14	0.125	0.470	-0.70	0.810	1	-3.84	0.113	1
hsa-miR-130a-3p	-3.08	0.147	0.513	2.40	0.428	1	-0.67	0.786	1

hsa-miR-152-3p	-2.18	0.266	0.708	-2.03	0.471	1	-4.21	0.074	1
**hsa-miR-25-3p**	**-4.25**	**0.005**	0.152	4.08	0.053	1	-0.17	0.921	1
**hsa-miR-206**	**1.61**	**0.011**	0.219	**-2.08**	**0.023**	0.874	-0.46	0.522	1
**hsa-miR-433-3p**	**-3.48**	**0.013**	0.221	3.53	0.075	1	0.05	0.973	1
**hsa-miR-425-5p**	**-5.59**	**0.024**	0.235	2.04	0.555	1	-3.56	0.213	1
**hsa-miR-186-5p**	**-4.67**	**0.046**	0.293	1.85	0.573	1	-2.82	0.299	1
**hsa-miR-873-5p**	-1.60	0.292	0.743	-2.29	0.296	1	**-3.89**	**0.035**	0.911
**hsa-miR-28-5p**	-1.32	0.384	0.804	-2.50	0.254	1	**-3.82**	**0.038**	0.911
**hsa-miR-625-3p**	-0.61	0.506	0.945	**3.39**	**0.014**	0.823	**2.78**	**0.014**	0.911
**hsa-miR-139-5p**	0.28	0.734	1	1.77	0.139	1	**2.04**	**0.040**	0.911

Supplementary Table S2:

Below the list of miRNA resulted significantly dysregulated in CI vs. CP vs. Ctrl in the Female subgroup of subjects (each set of data has been represented by p-value and ΔCq_{norm})

	CI vs CP (F)		CI vs Ctrl (F)		CP vs Ctrl (F)	
miRNA	p-value	ΔCq_{norm}	p-value	ΔCq_{norm}	p-value	ΔCq_{norm}
hsa-miR-17-5p	0.0022	-6.94	0.0378	-7.89	0.7036	-0.95
hsa-miR-199a-3p	0.0097	-7.59	0.1662	-7.54	0.5214	0.06
hsa-miR-7-5p	0.0140	-8.00	0.0400	-8.97	0.7877	-0.97
hsa-miR-584-5p	0.0199	-6.68	0.1583	-7.62	0.5008	-0.94
hsa-miR-152-3p	0.0200	-5.97	0.1609	-5.23	0.9460	0.74
hsa-miR-151a-3p	0.0234	-6.36	0.3688	-3.97	0.3449	2.39
hsa-miR-409-3p	0.0256	-5.32	0.0345	-6.63	0.8883	-1.31
hsa-miR-15a-5p	0.0301	-7.73	0.0599	-10.22	1	-2.49
hsa-miR-191-5p	0.0379	-6.51	0.2397	-7.44	0.9000	-0.93
hsa-miR-21-5p	0.0430	-6.94	0.0187	-11.47	0.4182	-4.53
hsa-miR-125b-5p	0.0460	-4.28	0.0102	-8.45	0.1013	-4.18
hsa-miR-221-3p	0.0467	-9.65	0.0599	-12.06	0.1893	-2.41
hsa-miR-16-5p	0.0571	-6.47	0.1095	-7.67	0.2964	-1.20
hsa-miR-223-3p	0.0683	-6.48	0.0396	-9.66	0.0821	-3.18
hsa-miR-505-3p	0.0685	-5.18	0.0208	-9.90	0.4126	-4.72
hsa-miR-25-3p	0.0811	-1.01	0.0333	-2.05	0.2964	-1.05
hsa-miR-205-5p	0.0892	-4.61	0.0208	-9.34	0.3336	-4.74
hsa-miR-130a-3p	0.1173	-3.98	0.0150	-8.87	0.1013	-4.89
hsa-miR-24-3p	0.1293	-5.29	0.0649	-7.79	0.4192	-2.51
hsa-miR-342-3p	0.1293	-2.39	0.1095	-5.42	0.8927	-3.03
hsa-miR-4286	0.1348	-5.49	0.0287	-10.47	0.1039	-4.97
hsa-miR-425-5p	0.1767	-5.75	0.0888	-9.30	0.0589	-3.55
hsa-miR-30b-5p	0.1797	-4.46	0.0400	-9.65	0.3336	-5.19
hsa-miR-192-5p	0.1838	-4.78	0.0888	-8.70	0.2225	-3.91
hsa-miR-485-3p	0.2024	-1.76	0.0345	-5.00	0.1907	-3.24
hsa-miR-92b-3p	0.2899	-4.84	0.0518	-10.79	0.1013	-5.95
hsa-miR-150-5p	0.3114	-0.56	0.3833	-0.53	0.9000	0.03
hsa-miR-625-3p	0.5294	-0.75	0.0345	-6.09	0.0484	-5.34
hsa-miR-93-5p	0.8378	-0.36	0.2486	-5.28	0.2746	-4.93
hsa-miR-142-3p	0.9680	-1.85	0.4888	-4.57	0.5894	-2.72
hsa-miR-133a-3p	1	0.24	0.0150	-7.79	0.0204	-8.03

Supplementary Table S3: CI vs CP vs Ctrl in male subjects

Below the list of miRNA resulted significantly dysregulated in CI vs. CP vs. Ctrl in the Males subgroup of subjects (each set of data has been represented by p-value and ΔCq_{norm})

	CI vs CP (M)		CI vs Ctrl (M)		CP vs Ctrl (M)	
miRNA	p-value	ΔCq_{norm}	p-value	ΔCq_{norm}	p-value	ΔCq_{norm}
hsa-miR-17-5p	0.4535	-3.58	1	0.68	0.5734	4.26
hsa-miR-199a-3p	0.1786	-8.13	1	2.46	0.2043	10.59
hsa-miR-7-5p	0.7541	-1.56	0.5637	3.60	0.1773	5.16
hsa-miR-584-5p	0.2273	-5.64	0.2703	6.94	0.0259	12.58
hsa-miR-152-3p	0.7541	-0.59	0.5637	4.43	0.1773	5.02
hsa-miR-151a-3p	0.3028	-4.59	0.5637	2.77	0.0754	7.36
hsa-miR-409-3p	0.2791	-3.01	NA	0.00	0.3605	3.01
hsa-miR-15a-5p	0.0560	-7.78	0.5821	-3.40	0.3706	4.39
hsa-miR-191-5p	0.2835	-3.92	0.2703	6.07	0.0455	9.99
hsa-miR-21-5p	0.4347	-2.03	0.1187	10.47	0.0455	12.50
hsa-miR-125b-5p	0.5310	-2.46	0.8255	-2.14	1	0.32
hsa-miR-221-3p	0.4263	-5.36	0.8454	3.23	0.1394	8.59
hsa-miR-16-5p	0.0080	-7.69	0.2286	-5.54	0.0769	2.15
hsa-miR-223-3p	0.0132	-11.29	0.1084	-8.12	0.1119	3.16
hsa-miR-505-3p	0.1174	-5.78	NA	0.00	0.1773	5.78
hsa-miR-25-3p	0.1786	-8.44	0.5926	-6.21	0.1119	2.23
hsa-miR-205-5p	0.7679	0.96	0.5637	3.47	0.5017	2.52
hsa-miR-130a-3p	0.1840	-4.64	NA	0.00	0.2562	4.64
hsa-miR-24-3p	0.0758	-10.69	0.5637	3.80	0.0137	14.49
hsa-miR-342-3p	0.9436	2.33	0.0436	14.10	0.0455	11.76
hsa-miR-4286	0.5971	-4.33	0.5637	3.87	0.1183	8.20
hsa-miR-425-5p	0.9434	-4.22	0.2703	7.08	0.0259	11.30
hsa-miR-30b-5p	0.1840	-4.57	NA	0.00	0.2562	4.57
hsa-miR-192-5p	0.0415	-8.62	NA	0.00	0.0754	8.62
hsa-miR-485-3p	0.6353	-0.78	NA	0.00	0.7150	0.78
hsa-miR-92b-3p	0.0415	-9.91	NA	0.00	0.0754	9.91
hsa-miR-150-5p	0.0394	-6.91	0.3590	-3.37	0.6116	3.54
hsa-miR-625-3p	NA	0.00	NA	0.00	NA	0.00
hsa-miR-93-5p	0.0415	-8.74	NA	0.00	0.0754	8.74
hsa-miR-142-3p	0.0962	-6.82	0.5637	2.83	0.0455	9.65
hsa-miR-133a-3p	0.0717	-6.33	NA	0.00	0.1183	6.33

Supplementary Table S4:

Spearman correlation test between all subjects and MMSE (data adjusted for age and sex).

miRNA	Spearman's rho	p-value	FDR
hsa-miR-223-3p	0.49	0.0017	0.074
hsa-miR-16-5p	0.49	0.0019	0.074
hsa-miR-433-3p	0.44	0.0057	0.1185
hsa-miR-151b	0.44	0.0060	0.1185
hsa-miR-15a-5p	0.41	0.0102	0.1516
hsa-miR-221-3p	0.41	0.0115	0.1516
hsa-miR-191-5p	0.39	0.0146	0.1554
hsa-miR-106b-5p	0.39	0.0165	0.1554
hsa-miR-199a-3p	0.38	0.0189	0.1554
hsa-miR-22-3p	0.38	0.0197	0.1554
hsa-miR-128-3p	0.37	0.0241	0.1724
hsa-miR-17-5p	0.36	0.0262	0.1724
hsa-miR-222-3p	0.35	0.0295	0.1793
hsa-miR-26a-5p	0.34	0.0352	0.1987
hsa-miR-505-3p	0.33	0.0441	0.2182
hsa-miR-192-5p	0.32	0.0468	0.2182
hsa-miR-335-5p	0.32	0.0472	0.2182

Supplementary Table S5: list of the 30 miRNAs downregulated in CI compared to CP subjects and the correspondent citations in other neuropsychiatric topics (cited in Table 2 of the main text, **references below**)*;* the third column indicates the consistency – if any - between our data and the in the last column, the comparison with our data (C= concordant; NC= not concordant, i.e. overexpressed)

**miRNA ID**		
hsa-miR-125b-5p	AD (Subasinghe K, Cells 2025)(Kumar A, Alzheimer's & Dementia 2023)	**C + NC**
hsa-miR-223-3p	Anxiety and depression (Wang W, Psychopharmacology 2025)	C
hsa-miR-15a-5p	Aging and cognitive decline (Ye Z, Peer J 2020)	C
hsa-miR-409-3p	Aging stem cells (Mi N, Curr Stem Cell Res Ther 2025)	C
hsa-miR-22-3p	AD (Liu S, J Neuroinflammation 2022)	C
hsa-miR-505-3p	Aging (Streese L, Sci Rep 2022)	**NC**
hsa-miR-106b-5p	AD (Kumar A, Alzheimer's & Dementia 2023)	**NC**
hsa-miR-30b-5p	MS (Groen K, BMC Ned Genomics 2018)	C
hsa-miR-16-5p	AD (Alamro H, Sci Rep 2023)	C
hsa-miR-130a-3p	AD (Wang Y, Am J Alzheimers Dis Other Demen 2021)	C
hsa-miR-17-5p	AD (Giuliani A, Front Aging Neurosci 2021)	**NC**
hsa-miR-335-5p	AD (Wang et al, Curr Neurovasc Res 2020)	C
hsa-miR-92b-3p	Cognitive domains (Melas K, Alzheimers Dement 2024)	C
hsa-miR-26a-5p	AD (Gascon E, Biomolecules 2024)	C
hsa-miR-192-5p	Cognition in PD (Guevremont D, Parkinson Relat Disord 2026)	C
hsa-miR-222-3p	*Cognitive functions in musicians (Nair PS, Peer J 2019)*	**NC**
hsa-miR-221-3p	Neuronal apoptosis (Pan, Histol Histopathol 2023)	C
hsa-miR-7-5p	AD (Gu Y, Curr Neurovasc Resc 2024)	C
hsa-miR-328-3p	MDD, ADHD (Honorato-Mauer, Transl Psichiatry 2023)	**NC**
hsa-miR-532-3p	*Experimental cognition (Liu Y, Psychopharmacology 2023)*	C
hsa-miR-128-3p	AD (Hashemi KS, Front Genet 2023)	C
hsa-miR-199a-3p	AD (Wang C, BMC Neurosci 2025)	C
hsa-miR-361-5p	*Music listening (Nair PS, Epigenetics 2021)*	C
hsa-miR-21-5p	AD (Garcia G, Cells 2022)	**NC**
hsa-miR-140-3p	AD (Song C, J Neuroinflammation 2024)	C
hsa-miR-24-3p	AD (Ansari A, Neurobiol Aging 2019)	C
hsa-miR-152-3p	AD (Lugli G, PloSOne 2015)	C
hsa-miR-584-5p	Stroke (Fernandez-Perez I, Neurology 2025)	**NC**
hsa-miR-191-5p	AD (Hong H, J Alzheimers Dis 2017)	C
hsa-miR-151a-3p	AD (Gamez-Valero, Transl Neurogener 2019)	C

Legend: CP= cognitively preserved; CI= cognitively impaired; AD= Alzheimer's Disease; MS= Multiple Sclerosis; MDD= Major Depressive Disorders; ADHD= Attention Deficit Hyperactive Disorders.

References to Supplementary Table S5:

1. Subasinghe K, Hall C, Rowe M, Zhou Z, Barber R, Phillips N. Neuronal Enriched Extracellular Vesicle miR-122-5p as a Potential Biomarker for Alzheimer's Disease. Cells. 2025 Nov 13;14(22):1784. doi: 10.3390/cells14221784.
2. Wang W, Yang T, Liu N, Yang L, Liu C, Qi X, Wang N, Wang M, Wang Y. MiR-223-3p inhibits hippocampal neurons injury and exerts anti- anxiety/depression-like behaviors by directly targeting NLRP3. Psychopharmacology (Berl). 2025 Aug;242(8):1797-1809. doi: 10.1007/s00213-025-06763-5.
3. Ye Z, Sun B, Mi X, Xiao Z. Gene co-expression network for analysis of plasma exosomal miRNAs in the elderly as markers of aging and cognitive decline. PeerJ. 2020 Jan 6;8:e8318. doi: 10.7717/peerj.8318.
4. Mi N, Liu X, Gao Y, Bai C, Li X. Probing the Mesenchymal Stem Cell Aging through *In silico* Assessment of Extracellular Vesicle-mediated miRNAs. Curr Stem Cell Res Ther. 2025;20(8):882-914. doi: 10.2174/011574888X342545241202050636.
5. Liu S, Fan M, Xu JX, Yang LJ, Qi CC, Xia QR, Ge JF. Exosomes derived from bone-marrow mesenchymal stem cells alleviate cognitive decline in AD-like mice by improving BDNF-related neuropathology. J Neuroinflammation. 2022 Feb 7;19(1):35. doi: 10.1186/s12974-022-02393-2
6. Streese L, Demougin P, Iborra P, Kanitz A, Deiseroth A, Kröpfl JM, Schmidt-Trucksäss A, Zavolan M, Hanssen H. Untargeted sequencing of circulating microRNAs in a healthy and diseased older population. Sci Rep. 2022 Feb 22;12(1):2991. doi: 10.1038/s41598-022-06956-4.
7. Kumar A, Su Y, Sharma M, Singh S, Kim S, Peavey JJ, Suerken CK, Lockhart SN, Whitlow CT, Craft S, Hughes TM, Deep G. MicroRNA expression in extracellular vesicles as a novel blood-based biomarker for Alzheimer's disease. Alzheimers Dement. 2023 Nov;19(11):4952-4966. doi: 10.1002/alz.13055.
8. Groen K, Maltby VE, Lea RA, Sanders KA, Fink JL, Scott RJ, Tajouri L, Lechner-Scott J. Erythrocyte microRNA sequencing reveals differential expression in relapsing-remitting multiple sclerosis. BMC Med Genomics. 2018 May 21;11(1):48. doi: 10.1186/s12920-018-0365-7.
9. Alamro H, Thafar MA, Albaradei S, Gojobori T, Essack M, Gao X. Exploiting machine learning models to identify novel Alzheimer's disease biomarkers and potential targets. Sci Rep. 2023 Mar 27;13(1):4979. doi: 10.1038/s41598-023-30904-5
10. Wang Y, Shi M, Hong Z, Kang J, Pan H, Yan C. MiR-130a-3p Has Protective Effects in Alzheimer's Disease via Targeting DAPK1. Am J Alzheimers Dis Other Demen. 2021 Jan-Dec;36:15333175211020572. doi: 10.1177/15333175211020572
11. Giuliani A, Gaetani S, Sorgentoni G, Agarbati S, Laggetta M, Matacchione G, Gobbi M, Rossi T, Galeazzi R, Piccinini G, Pelliccioni G, Bonfigli AR, Procopio AD, Albertini MC, Sabbatinelli J, Olivieri F, Fazioli F. Circulating Inflamma-miRs as Potential Biomarkers of Cognitive Impairment in Patients Affected by Alzheimer's Disease. Front Aging Neurosci. 2021 Mar 11;13:647015. doi: 10.3389/fnagi.2021.647015
12. Wang D, Fei Z, Luo S, Wang H. MiR-335-5p Inhibits β-Amyloid (Aβ) Accumulation to Attenuate Cognitive Deficits Through Targeting c-jun-N-terminal Kinase 3 in Alzheimer's Disease. Curr Neurovasc Res. 2020;17(1):93-101. doi: 10.2174/1567202617666200128141938.

13. Melas K, Talevi V, Imtiaz MA, Etteldorf R, Estrada S, Krüger DM, Pena-Centeno T, Aziz NA, Fischer A, Breteler MMB. Blood-derived microRNAs are related to cognitive domains in the general population. Alzheimers Dement. 2024 Oct;20(10):7138-7159. doi: 10.1002/alz.14197.
14. Gascón E, Calvo AC, Molina N, Zaragoza P, Osta R. Identifying Hub Genes and miRNAs Associated with Alzheimer's Disease: A Bioinformatics Pathway to Novel Therapeutic Strategies. Biomolecules. 2024 Dec 20;14(12):1641. doi: 10.3390/biom14121641.
15. Guévremont D, Roy J, Garvey A, McLean C, Cutfield N, Williams J. Plasma microRNA predict cognitive decline in Parkinson's disease. Parkinsonism Relat Disord. 2026 Feb;143:108149. doi: 10.1016/j.parkreldis.2025.108149.
16. Nair PS, Kuusi T, Ahvenainen M, Philips AK, Järvelä I. Music-performance regulates microRNAs in professional musicians. PeerJ. 2019 Mar 29;7:e6660. doi: 10.7717/peerj.6660
17. Pan J, Wu T, Chen B, Wu H. Exosomes derived from endothelial progenitor cells ameliorate glyoxylate deprivation (OGD)-induced neuronal apoptosis by delivering miR-221-3p. Histol Histopathol. 2023 Apr;38(4):423-430. doi: 10.14670/HH-18-528.
18. Gu Y, Chen N, Zhu L, Chen X, Jiang T, Zhang Y. Whole Transcriptome Sequencing of Peripheral Blood Identifies the Alzheimer's Disease-Related circRNA-miRNA-lncRNA Pathway. Curr Neurovasc Res. 2024;21(2):184-197. doi: 10.2174/0115672026305417240209062508
19. Honorato-Mauer J, Xavier G, Ota VK, Chehimi SN, Mafra F, Cuóco C, Ito LT, Ormond R, Asprino PF, Oliveira A, Bugiga AVG, Torrecilhas AC, Bressan R, Manfro GG, Miguel EC, Rohde LA, Pan PM, Salum GA, Pellegrino R, Belangero S, Santoro ML. Alterations in microRNA of extracellular vesicles associated with major depression, attention-deficit/hyperactivity and anxiety disorders in adolescents. Transl Psychiatry. 2023 Feb 6;13(1):47. doi: 10.1038/s41398-023-02326-4.
20. Liu Y, Shao J, Qiao R, Li J, Li C, Cao W. Xanthohumol improves cognitive impairment by regulating miRNA-532-3p/Mpped1 in ovariectomized mice. Psychopharmacology (Berl). 2023 May;240(5):1169-1178. doi: 10.1007/s00213-023-06355-1
21. Hashemi KS, Aliabadi MK, Mehrara A, Talebi E, Hemmati AA, Rezaeiye RD, Ghanbary MJ, Motealleh M, Dayeri B, Alashti SK. A meta-analysis of microarray datasets to identify biological regulatory networks in Alzheimer's disease. Front Genet. 2023 Aug 29;14:1225196. doi: 10.3389/fgene.2023.1225196.
22. Wang C, Bu X, Cao M, Lian Y, Ling H, You M, Yi J, Gao X, Wu D, Li Y. Mir-199a-3p aggravates neuroinflammation in an Alzheimer's disease transgenic mouse model by promoting M1-polarization microgliaMir-199a-3p M1. BMC Neurosci. 2025 Jul 24;26(1):45. doi: 10.1186/s12868-025-00965-5. Erratum in: BMC Neurosci. 2025 Sep 15;26(1):58. doi: 10.1186/s12868-025-00974-4.
23. Nair PS, Raijas P, Ahvenainen M, Philips AK, Ukkola-Vuoti L, Järvelä I. Music-listening regulates human microRNA expression. Epigenetics. 2021 May;16(5):554-566. doi: 10.1080/15592294.2020.1809853
24. Garcia G, Pinto S, Ferreira S, Lopes D, Serrador MJ, Fernandes A, Vaz AR, Mendonça A, Edenhofer F, Malm T, Koistinaho J, Brites D. Emerging Role of miR-21-5p in Neuron-Glia Dysregulation and Exosome Transfer Using Multiple Models of Alzheimer's Disease. Cells. 2022 Oct 26;11(21):3377. doi: 10.3390/cells11213377

25. Song C, Li S, Mai Y, Li L, Dai G, Zhou Y, Liang X, Zou OM, Wang Y, Zhou L, Liu J, Zou Y. Dysregulated expression of miR-140 and miR-122 compromised microglial chemotaxis and led to reduced restriction of AD pathology. J Neuroinflammation. 2024 Jul 2;21(1):167. doi: 10.1186/s12974-024-03162-z.
26. Ansari A, Maffioletti E, Milanesi E, Marizzoni M, Frisoni GB, Blin O, Richardson JC, Bordet R, Forloni G, Gennarelli M, Bocchio-Chiavetto L; PharmaCog Consortium. miR-146a and miR-181a are involved in the progression of mild cognitive impairment to Alzheimer's disease. Neurobiol Aging. 2019 Oct;82:102-109. doi: 10.1016/j.neurobiolaging.2019.06.005
27. Lugli G, Cohen AM, Bennett DA, Shah RC, Fields CJ, Hernandez AG, Smalheiser NR. Plasma Exosomal miRNAs in Persons with and without Alzheimer Disease: Altered Expression and Prospects for Biomarkers. PLoS One. 2015 Oct 1;10(10):e0139233. doi: 10.1371/journal.pone.0139233
28. Fernández-Pérez I, Vallverdú-Prats M, Rey-Álvarez L, Giralt Steinhauer E, Ois A, Cuadrado-Godia E, Rodriguez-Campello A, Suárez-Pérez A, Macias-Gómez A, Soriano-Tárraga C, Purroy FF, Arque G, Tur S, Cañellas G, Vives-Bauza C, Segura T, Serrano-Heras G, Lazcano U, Jiménez-Balado J, Jimenez-Conde J. Circulating miRNAs Associated With 3-Month Outcome in Patients With Acute Ischemic Stroke. Neurology. 2025 Jan 14;104(1):e210085. doi: 10.1212/WNL.0000000000210085
29. Hong H, Li Y, Su B. Identification of Circulating miR-125b as a Potential Biomarker of Alzheimer's Disease in APP/PS1 Transgenic Mouse. J Alzheimers Dis. 2017;59(4):1449-1458. doi: 10.3233/JAD-170156
30. Gámez-Valero A, Campdelacreu J, Vilas D, Ispierto L, Reñé R, Álvarez R, Armengol MP, Borràs FE, Beyer K. Exploratory study on microRNA profiles from plasma-derived extracellular vesicles in Alzheimer's disease and dementia with Lewy bodies. Transl Neurodegener. 2019 Oct 3;8:31. doi: 10.1186/s40035-019-0169-5

Supplementary Table S7: Details of comparison between EV and plasma pairs (Wilcoxon's test). The last 3 columns represent the number of pairs with $\Delta Cq_{norm} \geq 1$, $\Delta Cq_{norm} \leq 1$, $-1 \leq \Delta Cq_{norm} \leq 1$, respectively (as represented in Figure 4 of the main test).

miRNA ID	Wilcoxon's paired test			pairs	pairs with $\Delta Cq_{norm} \geq 1$	pairs with $\Delta Cq_{norm} \leq -1$	pairs with $-1 < \Delta Cq_{norm} < 1$
	p-value	FDR	ΔCq_{norm}				
hsa-miR-584-5p	0.0003	0.0028	3.8	17	17	0	0
hsa-miR-148b-3p	0.0003	0.0028	3.0	17	17	0	0
hsa-miR-185-5p	0.0003	0.0028	3.1	17	16	0	1
hsa-miR-24-3p	0.0004	0.0028	3.7	17	16	0	1
hsa-miR-145-5p	0.0004	0.0028	3.5	17	16	0	1
hsa-miR-25-3p	0.0003	0.0028	2.2	17	15	0	2
hsa-miR-186-5p	0.0005	0.0028	3.3	17	15	1	1
hsa-miR-15a-5p	0.0005	0.0028	3.2	17	15	0	2
hsa-miR-425-5p	0.0005	0.0028	3.6	16	15	0	1
hsa-miR-199a-3p	0.0003	0.0028	2.0	17	14	0	3
hsa-miR-484	0.0005	0.0028	2.8	16	14	0	2
hsa-miR-328-3p	0.0007	0.0035	2.7	16	14	0	2
hsa-miR-335-5p	0.0006	0.0031	2.5	16	13	0	3
hsa-miR-16-5p	0.0009	0.0036	2.4	16	13	1	2
hsa-miR-128-3p	0.0018	0.0059	3.3	16	13	1	2
hsa-miR-150-5p	0.0021	0.0067	2.7	16	13	1	2
hsa-miR-221-3p	0.0029	0.0087	1.6	17	13	1	3
hsa-miR-451a	0.0041	0.0099	2.3	15	13	1	1
hsa-miR-361-5p	0.0059	0.0130	3.1	15	13	1	1
hsa-miR-223-3p	0.0245	0.0435	2.3	16	13	2	1
hsa-miR-3615	0.0009	0.0036	2.0	16	12	0	4
hsa-miR-151b	0.0011	0.0041	1.9	17	12	1	4
hsa-miR-20a-5p	0.0039	0.0099	1.3	17	12	1	4
hsa-miR-93-5p	0.0042	0.0099	2.8	13	12	1	0
hsa-miR-140-3p	0.0011	0.0041	3.3	14	11	0	3
hsa-miR-151a-3p	0.0016	0.0058	3.0	15	11	0	4
hsa-miR-1260b	0.0039	0.0099	3.5	11	10	0	1
hsa-miR-22-3p	0.0051	0.0117	3.7	11	10	0	1
hsa-miR-21-5p	0.0068	0.0145	2.9	12	10	1	1
hsa-miR-532-3p	0.0084	0.0173	1.9	14	10	1	3
hsa-miR-222-3p	0.0098	0.0195	1.9	15	10	2	3
hsa-miR-205-5p	0.0039	0.0099	-4.0	11	0	11	0
hsa-miR-28-5p	0.0254	0.0439	-1.3	12	1	7	4
hsa-miR-424-5p	0.0591	0.0902	0.7	16	6	1	9
hsa-miR-505-3p	0.4561	0.4948	0.1	12	2	2	8
hsa-miR-192-5p	0.3936	0.4490	0.5	16	5	4	7
hsa-miR-338-3p	0.2093	0.2679	1.2	14	4	3	7
hsa-miR-1271-5p	0.1183	0.1578	0.5	15	6	2	7
